# Pathogenic variants identified by whole-exome sequencing in 43 patients with epilepsy

**DOI:** 10.1186/s40246-020-00294-0

**Published:** 2020-12-07

**Authors:** Linlin Zhang, Jinshuang Gao, Hailiang Liu, Yuan Tian, Xiaoli Zhang, Wei Lei, Ying Li, Yaqing Guo, Haiyang Yu, Erfeng Yuan, Lisi Liang, Shihong Cui, Xiaoan Zhang

**Affiliations:** 1 Clinical Laboratory, The Third Affiliated Hospital of Zhengzhou University, Zhengzhou, Henan 450052 People’s Republic of China; 2 Henan Academician Workstation of Genetic Diagnosis and Precision Medicine, Zhengzhou, Henan People’s Republic of China; 3 CapitalBio Genomics Co., Ltd., Dongguan, 532808 Guangdong People’s Republic of China; 4 Department of Neurologic Medicine, The Third Affiliated Hospital of Zhengzhou University, Zhengzhou, Henan 450052 People’s Republic of China; 5 Department of Obstetrics and Gynecology, The Third Affiliated Hospital of Zhengzhou University, 7 Front Kangfu Street, Zhengzhou, Henan 450052 People’s Republic of China; 6 Department of Obstetrics, The Third Affiliated Hospital of Zhengzhou University, Zhengzhou, Henan 450052 People’s Republic of China; 7 Department of Imaging, The Third Affiliated Hospital of Zhengzhou University, Zhengzhou, Henan 450052 People’s Republic of China; 8 Department of Imaging and Gynecologic Oncology, The Third Affiliated Hospital of Zhengzhou University, 7 Front Kangfu Street, Zhengzhou, Henan 450052 People’s Republic of China

**Keywords:** Epilepsy, Whole-exome sequencing (WES), Diagnostic yield, Pathogenic, De novo

## Abstract

**Background:**

Epilepsy is a group of neurological disorders characterized by recurrent epileptic seizures. Epilepsy is affected by many factors, approximately 20–30% of cases are caused by acquired conditions, but in the remaining cases, genetic factors play an important role. Early establishment of a specific diagnosis is important to treat and manage this disease.

**Methods:**

In this study, we have recruited 43 epileptic encephalopathy patients and the molecular genetic analysis of those children was performed by whole-exome sequencing (WES).

**Results:**

Fourteen patients (32.6%, 14/43) had positive genetic diagnoses, including fifteen mutations in fourteen genes. The overall diagnostic yield was 32.6%. A total of 9 patients were diagnosed as pathogenic mutations, including 4 variants had been reported as pathogenic previously and 6 novel variants that had not been reported previously. Therefore, WES heralds promise as a tool for clinical diagnosis of patients with genetic disease.

**Conclusion:**

Early establishment of a specific diagnosis, on the one hand, is necessary for providing an accurate prognosis and recurrence risk as well as optimizing management and treatment options. On the other hand, to unveil the genetic architecture of epilepsy, it is of vital importance to investigate the phenotypic and genetic complexity of epilepsy.

## Introduction

Epilepsy is a group of neurological disorders characterized by recurrent epileptic seizures. It affects about 3.5–6.5 per 1000 children [1] and 10.8 per 1000 elderly people [2], and 2 to 4 million new cases are diagnosed each year [3]. The characteristic phenotypic features of epilepsy are widely variable, such as infantile spasms, childhood absence epilepsy, and juvenile myoclonic epilepsy because of the ages, and intellectual disability (ID)/mental retardation (MR) is the most common comorbidity [4]. The onset of epilepsy is varied from infancy to adulthood [5]. Ohtahara syndrome (OS) is characterized that epileptic occurring in the first few months of life. Affected children may progress onto other epilepsy syndromes such as West syndrome, or they may die in infancy [6]. Meanwhile, individuals with epilepsy are reported to show clinical features of other disorders, or vice versa, such as attention-deficit/hyperactivity disorder are the most common comorbid conditions associated with epilepsy [1].

Epilepsy is affected by many factors [7], approximately 20–30% of cases are caused by acquired conditions such as head trauma and stroke, but in the remaining cases, genetic factors play an important role [8]. Hundreds of epilepsy genes have been identified, and many thousands of genes potentially involved in brain development and function [9]. The first reported for OS was the X-linked *ARX* gene, which encodes a developmental transcription factor. STXBP1, encoding syntax in binding protein 1, plays a role in exocytosis of synaptic vesicles and is emerging as one of the most important non-ion channel proteins responsible for a variety of neurodevelopmental disorders including early onset epileptic encephalopathy [10]. The *SCN1A* gene had previously been associated with epilepsy syndromes that are typically characterized by febrile seizures [11]. Approximately 60% of patients with classical lissencephaly have been found to have deletions or mutations of *PAFAH1B1*, which playing a role in both cellular division and motility, as well as the regulation of brain levels of platelet activating factor [12]. *TSC2* have been reported to be associated with tuberous sclerosis, which describe the saturation in some patients with seizures and mental subnormality [13]. Nearly 90 genes are currently known to be associated with the pathogenesis of early-onset infantile epileptic encephalopathy. However, many patients test negative in these reported genes. On the one hand, some genes have yet to be identified. Besides, mutations in reported genes are pathogenic, but the variants have not been reported previously, and it has not been listed in HGMD or ClinVar. Therefore, on the one hand, to give an early diagnosis for patients is necessary for providing an accurate prognosis and recurrence risk as well as optimizing management and treatment options. On the other hand, to unveil the genetic architecture of epilepsy is of vital importance to investigate the phenotypic and genetic complexity of epilepsy.

Epilepsy is a genetically heterogeneous disorder, and the genetic diagnosis of epilepsy is still challenging. While, with the rapid advance of NGS techniques, a number of rare disorders’ genetic basis have been revealed [14]. Thus, NGS has dramatically changed the way to mutation identification for rare diseases. WES is also a highly successful strategy to identify genetic changes, such as epileptic encephalopathy [15, 16]. It is useful clinically, on the one hand, and it can help make or confirm a diagnosis. On the other hand, it may allow counseling on recurrence risk and prenatal testing. In this study, we conducted a retrospective study to provide a contemporary account of the clinical epidemiology of newly diagnosed epilepsy as evaluated in The Third Affiliated Hospital of Zhengzhou University. In this study, we wanted to examine the use and yield of WES for molecular genetic of children with epileptic encephalopathies with unknown etiology and to provide more reference data for WES detection of epilepsy. Here, we report the outcome of WES for genetic diagnosis of cases with epilepsy including 9 cases of pathogenic variants, among which 6 were novel.

## Patients and methods

### Study design and participants

The 98 patients were transferred from 45 hospitals to The Third Affiliated Hospital of Zhengzhou University. After screening, a total of 43 epilepsy children with unknown cause were enrolled. Doctors opted WES to unveil the genetic architecture of this children. Parents of all patients signed informed consent, and the study passed the ethical approval of The Third Affiliated Hospital of Zhengzhou University. The inclusion criteria are as follows: (1) the main indication for testing was epilepsy of unknown source in patients in whom epilepsy was the primary disease phenotype, (2) multiple epileptiform discharges with severely disorganized background activity on electroencephalography (EEG), (3) having a family history of epilepsy, (4) progressive developmental deterioration or a known developmental and epileptic encephalopathy syndrome. The exclusion criteria are as follows: (1) metabolic abnormalities, (2) significant structural lesion detected on brain magnetic resonance imaging, (3) central nervous system infections, (4) hypoxic ischemic encephalopathy, and (5) abnormalities detected on previous genetic tests.

### Whole-exome sequencing

Whole blood collected in tubes with EDTA was accepted for testing. MagPure Genomic DNA Extraction Kit for Genomic DNA Extraction and Frag enzyme of NEB Corporation were used to perform fragmentation. Afterwards, end repair, connector connection, and the products were purified with Agencour AMPure XP Kit. SureSelect TE Reagent Kit, PTN, 16 (Agilent) kit was used to build the library, and the closed library was hybridized and captured for 20 h. Finally, sequencing was performed according to the protocols of JingXin BioelectronSeq 4000 System (CFDA registration permit NO. 20153400309) which is a kind of semiconductor sequencing platform.

### Variant filtering strategy

Sequence reads were mapped against the human reference genome (hg19) using BWA. Base quality recalibration, indel realignment, and variant calling were performed using GATK as previously described [17]. Two software tools (ForestDNM and mirTrios) were used for de novo mutation detection. Variants were annotated relative to RefSeq transcripts using ANNOVAR [18] software tool and in-house codes. Gene annotations were made against the UCSC Known Genes database. Previously known variants were annotated with their allele frequencies (using all ethnic affiliations) from the 1000 Genomes Project Feb. Non-synonymous variants were annotated with Polyphen2, MutationTaster, and EvoDscores. We then prioritized variants that were predicted to alter the protein sequence in any transcript (non-synonymous SNVs, stop loss or gain variants, indels or splice site mutations), particularly those that were highly conserved across the 46 vertebrate species in the UCSC conservation track. The consequences of mutations for protein products were inferred based on RefSeq gene annotations (http://www.ncbi.nlm.nih.gov/refseq/). Functional predictions for missense variants were obtained using 4 software tools: SIFT, LRT, SiPhy, and VEST3. Functional predictions for splicing site variants were obtained using spliceAI.

### Variant validation

All putatively causal variants were Sanger-sequenced to confirm the genotypes of the proband, parents, and other disease members with family history.

## Results

### Patients

A total of 43 patients met the inclusion criteria. Of the 43 probands, 24 were male and 19 were female. All probands were less than 18 years old at the time of WES, and the mean age at the time of WES was 3.45 ± 3.21 (Table 1).

**Table 1:** Characteristics of patients having whole-exome sequencing

Age	Patients, *n*	Male, *n*	Famale, *n*	Mean age at presentation (SD), years
0–1 years	9	6	4	0.56 ± 0.19
1–5 years	19	11	10	2.36 ± 1.17
5–18 years	11	7	5	7.78 ± 2.57
Total	43	24	19	3.45 ± 3.21

### Diagnostic rate of WES

Table 2 summarized the diagnostic rate of epileptic cases. Overall, a molecular diagnosis (with the causative variant(s) identified in a well-established clinical gene) was provided for 14 of the 43 total cases (32.6%). The breadth of molecular diagnoses was large. In these 14 cases, 5 were reported as likely pathogenic and 9 were reported as pathogenic variants. In addition, 5 of the 43 total cases (11.6%) had potential molecular diagnoses (Table 2). 20 of the 43 cases (46.5%) had no significant variant.

**Table 2:** Overall molecular diagnosis rate

	Total (***N*** = 43)	
	No. of patients	Patients’ rate (%)
Diagnosis	14	32.6
Potential diagnosis	5	11.6
No significant variant	24	55.8

### Case presentation

Collectively, 9 cases were diagnosed as pathogenic, and the other 5 cases were diagnosed as likely pathogenic. 15 variants spanning 14 genes were identified and classified as pathogenic or likely pathogenic in 43 patients. Among them, 8 (8/15, 53.3%) variants were novel or containing different amino acid change from previously reported pathogenic variants. Clinical information and molecular diagnosis of the pathogenic cases were shown in Tables 3 and 4.

**Table 3:** Clinical description of 9 patients identified with epilepsy

Number	Sex	Age	Clinical presentation	Other
1	M	5 months	Epileptic of unknown cause, infantile spasm, developmental delayed	
2	M	11 years	First episode at 9 months, secondary seizure,	Family history: Father, first seizure at 20 years; grandma’s brother of proband: twitching history
3	F	7 months	Epileptic of unknown cause, intermittent convulsion for 3 months, sometimes a daze	
4	F	7 years	Epileptic of unknown cause, developmental delayed, low muscle tone	
5	F	10 years	Epileptic of unknown cause for once, hypophrenia, epilepsy continued treatment, white spots on the body and red pimple on the face, suspected tuberous sclerosis	
6	F	1 year and 8 months	Epilepsy, limb spasm, and eyes turned up during seizures	
7	F	2 months and 12 days	intermittent convulsion for 1 week, sometimes more than ten times a day, abnormal electroencephalogram (EEG), MRI normal	Previous investigations included: MRI and EEG
8	F	1 years	Epileptic of unknown cause, intermittent convulsion for 3 years, developmental delayed	
9	M	6 months and 18 days	Seizures, infantile spasms, developmental delays, obesity	

*M* male, *F* female, *MRI* magnetic resonance imaging, *EEG* electroencephalogram

**Table 4:** Clinical description and genetic diagnosis of 9 patients identified with epilepsy

Case	Disease gene	Inheritance	Mutations and protein alteration	Clinical implications of genetic information	Novel/reported	Other variants
1	PAFAH1B1	De novo, AD	NM_000430 c.830A>C p.H277P	Lissencephaly 1 OMIM: 607432	Reported	
2	LGI1	Heterozygous, Paternal, AD	NM_000682 c.215+2T>A p.Ile187Met	Epilepsy, familial temporal lobe,1. OMIM: 600512	Novel	ADRA2B:NM_000682: c.561C>G: p.Ile187Met VUS
3	SCN1A	De novo, AD	NM_001165963 c.680T>G p.Ile227Ser	Dravet syndrome OMIM: 607208	Reported	
4	ATP1A3	De novo, AD	NM_001256213 c.2476G>A p.Glu826Lys	Alternating hemiplegia of childhood, OMIM: 614820	Novel	
5	TSC2	De novo, AD	NM_000548.4:c.226-2A>G	Tuberous sclerosis 2 OMIM:191100	Reported	
6	PCDH19	De novo, XL	NM_001184880.1:c.1605_1612del:p.K536fs	Epileptic encephalopathy, early infantile, 9 OMIM:300088	Novel	
7	STXBP1	De novo, AD	NM_003165.3:c.429+1G>C	Epileptic encephalopathy, early infantile, 4 OMIM: 612164	Novel	SET:NM_001122821.1: c.746A>G:p.D249G
8	FGF12	De novo, AD	NM_021032.4:c.341G>A,P	Epileptic encephalopathy, early infantile, 47 OMIM:617166	Reported	
9	GABRB3	De novo, AD	NM_000814.6:c.154C>G,P	Epileptic encephalopathy, early infantile, 43 OMIM:617113	Novel	

*AD* autosomal dominant, *XL* X-linked

The pathogenic mutations carried by 4 patients have reported previously: case 1, case 3, case 5, and case 8. The clinical information and molecular diagnosis was shown in Tables 3 and 4. All of the patients’ analysis identified a de novo heterozygous missense variant except case 2. Case 1 identified a mutation in *PAFAH1B1* (NM_000430:c.830A>C:p.His277Pro) and was diagnosed as Lissencephaly (LIS). Case 3 identified a mutation in *SCN1A* (NM_001165963:c.680T>G:p.Ile227Ser) and was diagnosed as Dravet syndrome (OMIM:607208). Case 5 identified a heterozygous mutation in *TSC2* and was diagnosed as tuberous sclerosis 2(OMIM: 191100). Case 8 identified a mutation in *FGF12* (NM_021032.4:c.341G>A: p.R114H). All of the variants had been reported previously as pathogenic in ClinVar.

Case 2 was a boy aged 11 years. He had a history of epilepsy and had a paroxysmal convulsion for 9 months. The boy’s father started seizures when 20 years old, and the grandma’s brother of proband has a twitching history. The mutation identified in this group included a heterozygous mutation, inherited from his father in *LGI1* (NM_005097.4:c.215+2T>A:p.Ile187Met). Mutation in *LGI1* could cause epilepsy, familial temporal lobe, 1 (OMIM:600512). It is characterized by partial seizures originating from the temporal lobe [19]. His family has a history of epilepsy and affected people include his father and grandfather (grandma’s brother of the proband). Patients in this family all possess this mutation (NM_005097.4: c.215+2T>A:p.Ile187Met), which was confirmed by Sanger sequencing (Supplementary figure). This splice-site mutation in *LGI1* could cause the length of the protein encoded by it be truncated or degraded. The variant was extremely rare, since it was not included in 1000 Genomes or NHLBI Exome Sequencing Project (ESP), and mutation was clearly predicted to be functionally deleterious based on mutation effect prediction tools. So the variant c.215+2T>A in *LGI1* was classified as pathogenic according to the above evidences and ACMG guidelines. The mutation was novel. In addition, the proband and his father have variant c.561C>G in *ADRA2B* (NM_000682.7) but the grandfather does not. So we classify this variant as an uncertain significance group.

Case 4 was a girl aged 7 years with epilepsy. She suffered from developmental delayed and low muscle tone. Her analysis identified a de novo heterozygous missense variant (NM_001256213.2:c.2476G>A:p.Glu826Lys) in *ATP1A3*, which was confirmed by Sanger sequencing. Mutation in *ATP1A3* cause alternating hemiplegia of childhood (AHC, OMIM:614820), which is a rare syndrome characterized by infantile onset of episodic hemi-or quadriplegia. Most cases are accompanied by dystonic posturing, choreoathetoid movements, abnormal ocular movements, developmental delay, and progressive cognitive impairment [20]. Several studies reported *ATP1A3* mutation could cause alternating hemiplegia of childhood (ACH) [21, 22], but these variants are extremely rare, and it is not included in 1000 Genomes, NHLBI Exome Sequencing Project (ESP), and ExAC. So the variant c.2476G>A in *ATP1A3* is classified as pathogenic according to the above evidences and ACMG guidelines. The mutation was novel.

Case 6 was a girl aged 1 year and 8 months. She suffered from epilepsy, limb spasm, and eyes turned up during seizures. She was detected with de novo heterozygous frameshift deletion variant in *PCDH19*, which was confirmed by Sanger sequencing. Mutation in *PCDH19* cause epileptic encephalopathy, early infantile, 9 (EIEE9, OMIM:300088), also known as epilepsy and mental retardation restricted to females (EFMR). EIEE9 is an X-linked disorder characterized by seizure onset in infancy and mild to severe mental retardation. Seizures are mainly tonic-clonic seizures, which are difficult to control [23]. The deletion mutation (NM_001184880.1:c.1605_1612del:p.K536fs) could cause a change in the reading frame, resulting in a change in protein function. The variant is not included in 1000 Genomes or NHLBI Exome Sequencing Project (ESP). It was classified as pathogenic according to the above evidences and ACMG/AMP guidelines. The mutation had not been reported previously and has not been listed in ClinVar, so it was novel.

Case 7 was a girl aged 2 months. She had intermittent convulsions for 1 week, sometimes more than ten times a day. However, there was no obvious abnormality in the MRI test, and the EEG test showed abnormal EEG, large number of multifocal slow waves and mixed waves. She was identified with *STXBP1* heterozygous splicing site variant and *SET* heterozygous missense variant. Both variants are de novo, which were confirmed by Sanger sequencing. The variants are rare, since they are not included in 1000 Genomes or NHLBI Exome Sequencing Project (ESP). The variant in *STXBP1* (NM_003165.6: c.429+1G>C) occurs in the splicing region and could lead to changes in protein function. Mutation in *STXBP1* could cause epileptic encephalopathy, early infantile, 4 (OMIM:612164) [24] via autosomal dominant, which is a severe form of epilepsy. It is characterized by frequent tonic seizures or spasms beginning in infancy, and our patient’s phenotype and inheritance pattern were consistent with epileptic encephalopathy, early infantile, 4. Therefore, it was classified as pathogenic according to the above evidences and ACMG/AMP guidelines, and the diagnosis can be made. The variant in *SET* (NM_001122821.1: c.746A>G: p.D249G) is evolutionarily conservative, but the protein function is tolerable predicted by SIFT. Mutation in *SET* cause mental retardation, autosomal dominant 58 (OMIM: 618106), which is a rare hereditary neurological disease characterized by early-onset cognitive impairment. The disease may be associated with autism, epilepsy and neuromuscular defects. Due to the young age of the patient, it is currently impossible to judge whether there is mental retardation. The variant in *SET* is classified as likely pathogenic according to the above evidences and ACMG guidelines. Both mutations were novel.

Case 9 was a boy aged 6 months 18 days with epilepsy, infantile spasms, developmental delay, and obesity. A de novo heterozygous missense variant (NM_000814.6: c.154C>G: p.L52V) was identified in *GABRB3*. And it was confirmed by Sanger sequencing method. Mutations in *GABRB3* can lead to autosomal dominant disease epileptic encephalopathy, early infantile, 43 (EIEE43, OMIM:617113). The main manifestations of EIEE43 are epilepsy, growth retardation, hyperactivity, mental disability, ataxia, systemic dystonia, infantile onset, dyskinesia, and epileptic encephalopathy [25, 26]. This variant occurs in the neurotransmitter-gated ion-channel ligand-binding domain. Mutations in this protein region may negatively impact on receptor function [25], and it might affect splicing predicted by spliceAI. This variant c.154C > G is a missense mutation of *GABRB3*, which causes EIEE43 through the mechanism of missense mutation. Being a very rare de novo variant, data on allelic frequency are not yet available. This variant in *GABRB3* is classified as pathogenic according to the above evidences and ACMG guidelines. And it was novel.

Lastly, 5 cases were reported as likely pathogenic shown in supplementary Table 1. Mutation of case 10 including a likely pathogenic variant in *HEXB* (NM_000521:c.841C>T:p.Arg281X), and two VUS variants inherited from his father. Mutation of case 11 included a likely pathogenic variant in *SYNGAP1* (NM_006772:c.1447_1448del:p.Phe484Pro fs*8). Mutation of case 12 included a homozygous variant in *AP4M1* (NM_004722:c.26C>T:p.Ser9Phe). Mutation of case 13 included a heterozygous variant in *NR2F1* (NM_005654.5:c.452 T>G:p.M151R) and case 14 included a homozygous variant in *ASNS* (NM_001673.4:c.146G>A:p.R49Q). See supplementary Table 1.The point of this study is to discuss the pathogenic cases, so these 5 patients are not the subjects we focus on.

## Discussion

Epilepsy is affected by many factors, and genetic factors play an important role. Genetic testing is important for children with epilepsy. Thus, it could explain the reason of epilepsy from the genetic aspect and make a contribution to unveil the genetic architecture of epilepsy. In addition, early establishment of a specific diagnosis is necessary for the management and treatment options. WES heralds promise as a tool for clinical diagnosis of patients with genetic disease. With the decreasing costs of WES, more patients with suspected genetic diseases will be affordable. Proband-only WES in recent studies had an overall diagnostic rate of 22 ~ 28.8% [27–29]. Several reports using a trio-based WES strategy have reported positive yields of 16–62% [27, 30]. Our results suggest that there is a substantial value of applying WES in patients with epilepsy of unknown etiology. The overall diagnostic yield was 32.6%, which falls within the reported range by others [31]. Of the 14 cases, 9 cases were reported as pathogenic variants, and 5 cases were reported as likely pathogenic. 9 de novo variants and 1 heterozygous paternal variant in these 9 cases. Affected genes include *PAFAH1B1, LGI1, SCN1A, ATP1A3, TSC2, STXBP1, SET, FGF12,* and *GABRB3.*

Mutations of case 1, 3, 5, and 8 were reported previously as pathogenic. All the 5 de novo variants were listed in ClinVar database, including c.830A>C in *PAFAH1B1*, c.680T>G in *SCN1A*, c.226-2A>G in *TSC2*, and c.341G>A in *FGF12*. A heterozygous came from his parents Dravet syndrome (DS) is a severe developmental and epileptic encephalopathy. DS is characterized by the onset of prolonged febrile and afebrile seizuresin infancy, and evolving to drug-resistant epilepsy with accompanying cognitive, behavioral, and motor impairment. Most cases are now known to be caused by pathogenic variants of gene *SCN1A* [32]. Both the genetic test and clinical phenotype of case 3 were consistent with DS, so the diagnosis was made. In addition, case 5 has had a seizure once and is currently in continuous treatment. Her genetic diagnosis confirmed a c.226-2A>G in *TSC2*. *TSC2* mutation could cause tuberous sclerosis, which is characterized by hamartomas in multiple organ systems, including the brain, skin, heart, kidneys, and lung. These changes can result in epilepsy, learning difficulties, behavioral problems, and renal failure, among other complications [13, 33].

Five candidate variants are found in genes not currently reported previously in 4 patients, c.215+2T>A in *LGI1*, c.2476G>A in *ATP1A3*, c.1605_1612del in *PCDH19*, c.429+1G>C in *STXBP1*, and c.154C>G in *GABRB3*.

*LGI1* has been reported associated with the etiology of epilepsy [34]. *LGI1* is located at a linkage region on chromosome 10q24. It encodes a member of the secreted leucine-rich repeat (LRR) superfamily, and it shares homology with members of the SLIT protein family. Mutations in *LGI1* are prevalent in autosomal dominant lateral temporal epilepsy (ADLTE) [35]. Variant of c.215+2T>A in *LGI1* was heterozygous inherited from his father in this study. The patient has a family history of epilepsy and affected people in this family all possess this mutation (NM_000682:c.215+2T>A:p.Ile187Met). So the diagnosis was made, and the mutation was novel that has not been reported previously.

ATP1A3 is a member of the gene family that encodes the alpha subunits of Na+/K+ transporting ATPase, and it is mainly expressed in interneurons and pyramidal cells, playing important roles in the brain. In AHC, *ATP1A3* mutations are distributed across the entire coding region. In the protein, mutations are located in highly conserved regions of the α3-subunit of the Na+/K+-ATPase and affect various functional domains and different transmembrane regions [36].

Mutation in *PCDH19* cause epileptic encephalopathy, early infantile, 9 (EIEE9, OMIM:300088), also known as epilepsy and mental retardation restricted to females (EFMR). The causative gene, *PCDH19*, is on the X-chromosome and encodes a cell-cell adhesion protein with restricted expression during brain development [37]. The deletion mutation c.1605_1612del could cause a change in the reading frame, resulting in a change in protein function. PCDH19-related epilepsy also known as epilepsy and mental retardation restricted to females (EFMR) is characterized by a distinctive pattern of X-linked inheritance, where heterozygous females exhibit seizures and hemizygous males are asymptomatic. Rakotomamonjy et al. observed the heightened susceptibility in Pcdh19 knockout females [38]. Therefore, women with early-onset epilepsy should pay special attention to the detection of the *PCDH19* gene. Therefore, special attention should be paid to the detection of *PCDH19* in female with early onset epilepsy.

The syntaxin binding protein 1 gene (*STXBP1*) is located on chromosome 9q34.11. Saitsu et al. identified de novo *STXBP1* mutations in five patients with Ohtahara syndrome [39]. Thereafter, many *STXBP1* mutations have been reported in patients with epileptic encephalopathy. Variant of c.429+1G>C in *STXBP1* is in the exon 6 splicing region which involved in constructing sec1-like domain 2. So the variant could lead to changes in protein function. The functionally impaired STXBP1 could affect synaptic function in the human brain [40]. Combined the clinical information and molecular diagnosis, we defined the variant as pathogenic mutation, which was novel.

In 2013, Epi et al. reported 4 unrelated patients with infantile epileptic encephalopathy. They concluded that their results implicated the *GABRB3* gene in epileptic encephalopathy [26]. Since then, approximately 30 cases have been described in subjects with different types of epileptic encephalopathies. The gene *GABRB3* encodes gamma-aminobutyric acid type A receptor beta-3, a member of the GABAA receptor gene family of heteromeric pentameric ligand-gated ion channels, which is deputed to mediate inhibitory signaling within the central nervous system [41]. The variant c.154C>G occurs in the neurotransmitter-gated ion-channel ligand-binding domain. Mutations in this protein region may negatively impact on receptor function [25]. P et al. described in the case report that the patient showed abundant panniculus adipose. Interestingly, besides suffering from epilepsy, case 9 was also obese. Arion et al. performed a microarray transcriptome profiling of anterolateral temporal cortical samples originating from individuals who suffered with temporal lobe epilepsy. They found that gene expression profiling revealed a downregulation of multiple GABA system-related genes (GABRA5, GABRB3, ABAT) in the spiking samples and an upregulation of lipid metabolism transcripts [42]. Therefore, we speculate that GABRB3 gene mutations in epilepsy patients might affect lipid metabolism.

Giving genetic testing to all children with epilepsy could yield many benefits, including ending the diagnostic odyssey during which parents and physicians spend untold amounts of time searching for an explanation for a child’s epilepsy and reduces associated costs [43], and creating the opportunity to participate in research studies of new therapies and to find optimal therapy and management approaches for the child or for others in the future.

## Conclusion

In this study, we have presented evidence suggesting that WES has great advantages for diagnosing epileptic of unknown cause. Early establishment of a specific diagnosis, on the one hand, is necessary for providing an accurate prognosis and recurrence risk as well as optimizing management and treatment options. On the other hand, to unveil the genetic architecture of epilepsy, it is of vital importance to investigate the phenotypic and genetic complexity of epilepsy. The application of this technology in the clinical setting still requires thorough investigation. Thus, more and more clinical research will be needed to support this technology in epilepsy.

## Supplementary Information

**Additional file 1: Supplementary table 1.** Clinical description and genetic diagnosis of 5 patients identified with epilepsy

**Additional file 2: Supplementary figure 1.** Sanger sequencing of family member in case 2.

## Data Availability

The datasets used and/or analyzed during the current study are available from the corresponding author on reasonable request.

## Abbreviations

WES: Whole-exome sequencing
NGS: Next-generation sequencing
OS: Ohtahara syndrome
ESP: Exome Sequencing Project
DS: Dravet syndrome
LRR: Leucine-rich repeat
AHC: Alternating hemiplegia of childhood
EEG: Electroencephalogram
