# Supplementary figures and images for: Pathogenic variants identified by whole-exome sequencing in 43 patients with epilepsy
Source: Hum Genomics. 2020 Dec 7;14:44. doi: 10.1186/s40246-020-00294-0 (PMC7720389; doi:10.1186/s40246-020-00294-0)

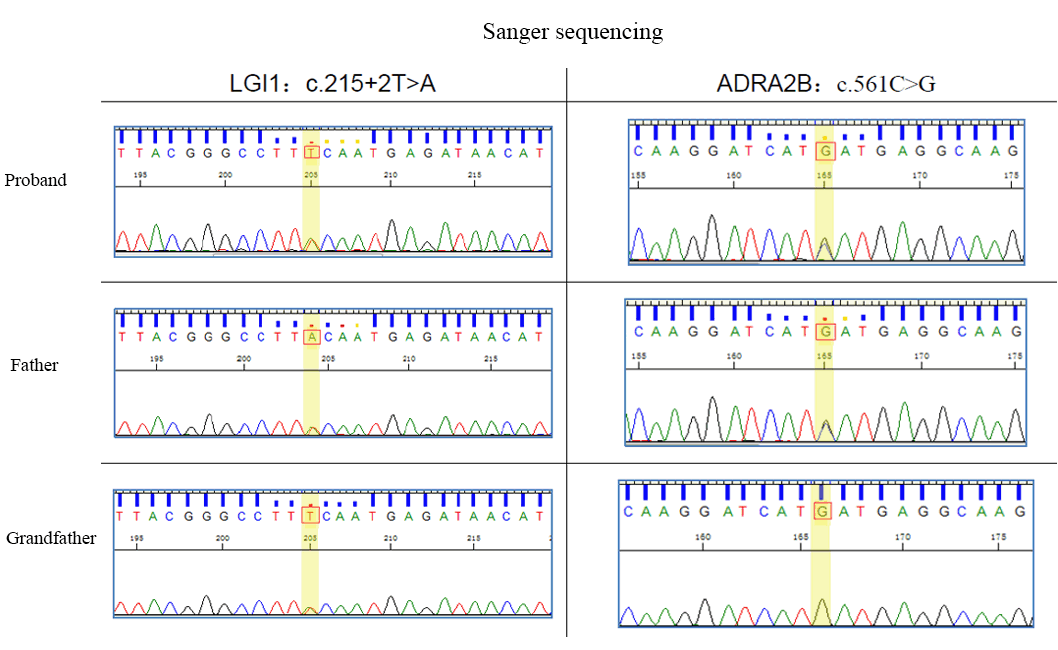

Supplement: Supplementary file 2 — Additional file 2: Supplementary figure 1. Sanger sequencing of family member in case 2. [file 40246_2020_294_MOESM2_ESM.gif]
